# Approaches to Brain Stress Testing: BOLD Magnetic Resonance Imaging with Computer-Controlled Delivery of Carbon Dioxide

**DOI:** 10.1371/journal.pone.0047443

**Published:** 2012-11-05

**Authors:** W. Alan C. Mutch, Daniel M. Mandell, Joseph A. Fisher, David J. Mikulis, Adrian P. Crawley, Olivia Pucci, James Duffin

**Affiliations:** 1 Department of Anesthesia and Perioperative Medicine, University of Manitoba, Winnipeg, Canada; 2 Department of Medical Imaging, Neuroradiology, University of Toronto, Toronto, Canada; 3 Department of Anesthesia, University of Toronto, Toronto, Canada; 4 Thornhill Research Inc., University of Toronto, Toronto, Canada; University of Queensland, Australia

## Abstract

**Background:**

An impaired vascular response in the brain regionally may indicate reduced vascular reserve and vulnerability to ischemic injury. Changing the carbon dioxide (CO_2_) tension in arterial blood is commonly used as a cerebral vasoactive stimulus to assess the cerebral vascular response, changing cerebral blood flow (CBF) by up to 5–11 percent/mmHg in normal adults. Here we describe two approaches to generating the CO_2_ challenge using a computer-controlled gas blender to administer: i) a square wave change in CO_2_ and, ii) a ramp stimulus, consisting of a continuously graded change in CO_2_ over a range. Responses were assessed regionally by blood oxygen level dependent (BOLD) magnetic resonance imaging (MRI).

**Methodology/Principal Findings:**

We studied 8 patients with known cerebrovascular disease (carotid stenosis or occlusion) and 2 healthy subjects. The square wave stimulus was used to study the dynamics of the vascular response, while the ramp stimulus assessed the steady-state response to CO_2_. Cerebrovascular reactivity (CVR) maps were registered by color coding and overlaid on the anatomical scans generated with 3 Tesla MRI to assess the corresponding BOLD signal change/mmHg change in CO_2_, voxel-by-voxel. Using a fractal temporal approach, detrended fluctuation analysis (DFA) maps of the processed raw BOLD signal per voxel over the same CO_2_ range were generated. Regions of BOLD signal decrease with increased CO_2_ (coded blue) were seen in all of these high-risk patients, indicating regions of impaired CVR. All patients also demonstrated regions of altered signal structure on DFA maps (Hurst exponents less than 0.5; coded blue) indicative of anti-persistent noise. While ‘blue’ CVR maps remained essentially stable over the time of analysis, ‘blue’ DFA maps improved.

**Conclusions/Significance:**

This combined dual stimulus and dual analysis approach may be complementary in identifying vulnerable brain regions and thus constitute a regional as well as global brain stress test.

## Introduction

The brain is exquisitely sensitive to alterations in carbon dioxide (CO_2_) tension in the blood.[1] Much time and effort is spent in neuroanesthesia and neurocritical care to control arterial CO_2_ tension because a higher CO_2_ increases intracranial pressure and volume. Increased CO_2_ in normal circumstances increases cerebral blood flow (CBF) by up to 5–11percent/mmHg [2] with a concomitant increase in cerebral blood volume. Alterations in this tight relationship between CO_2_ tension and CBF occur with brain injury and disease states. Frequently cerebrovascular reactivity (CVR) to CO_2_ is diminished or in certain circumstances actually reversed – a phenomenon termed ‘cerebral steal’.[3] [4] Patients with these alterations are at far greater risk of a poor outcome following surgical intervention; for example, stroke. Thus, manipulation of CO_2_ to prospectively assess patient risk of cerebral injury has been used for some time. These include increases in CO_2_ by breath-holding or administration of acetazolamide to increase intracellular CO_2_ and hydrogen ion concentration.[5] To date, blood velocity changes following CO_2_ challenge are routinely assessed by transcranial Doppler.[6] Improving the reproducibility and precision of the CO_2_ challenge for cerebral stress testing married to magnetic resonance imaging (MRI) for true regional assessment represents a significant step forward.[7] This objective, is in part, addressed in this study.

Higher field strength MRI using blood oxygen level dependent (BOLD) contrast has provided improved resolution in following changes in CBF and tissue oxygenation. Resting state [8] [9] and default mode [10] studies have helped define consciousness, and task-related BOLD imaging has demonstrated brain regional interconnectivity.[11] [12] Here we describe dynamic brain imaging in health and disease using carefully controlled alterations in CO_2_ to help understand CVR. We have used two patterns of changes in CO_2_ (a stepwise change in CO_2_ and a ramp change) and analyzed the resultant MR-BOLD output as a voxel-based ratio of change in mean BOLD signal to change in CO_2_ to determine regional CVR [13] - and a voxel-based fractal time analysis of the MR-BOLD signal using detrended fluctuation analysis (DFA).[14] The latter technique has been successfully applied to identify activated cortical areas with functional MRI. The similarities and differences between the two carefully controlled CO_2_ challenges and analysis paradigms are outlined. Further understanding of these relationships should advance the concept of a ‘brain stress test’ to aid in diagnosis and treatment of brain disease.

## Methods

All patients and volunteer subjects signed written informed consent with the various protocols approved by the Ethics Review Board of the University Health Network of the University of Toronto.

### Patient Group

Eight patients with severe carotid stenosis were screened by questionnaire for demographic data and any contraindication to undergoing MRI. Patients had been referred to the CVR study group because of significant cerebrovascular pathology. After familiarization with the protocol and fit to a breathing circuit (see below) the patients were placed in the bore of a 3.0 Tesla MR unit (Signa; GE Healthcare, Milwaukee, WI) and imaged per protocol with a BOLD echo planar sequence to assess the regional brain response to alterations in CO_2_ (see below).

*Volunteer Subject Group* – Two healthy volunteers were studied. After familiarization with the protocol they were fit to a breathing circuit (see below). They also had simultaneous bilateral recording of middle cerebral artery blood velocity by transcranial Doppler (Spencer Technologies, Seattle, WA), and bilateral frontal cerebral oximetry (Fore-Sight, CasMed, Branford, CT), continuous finger plethysmography (Nexfin, BMEYE, Amsterdam, The Netherlands), and continuous pulse oximetry (Nellcor, Covidien, Mansfield, MA). The hemodynamic responses to the changes in CO_2_ were recorded.

### Model-based prospective end-tidal (MPET) gas breathing sequences

The theory and application of the MPET approach (RespirAct - Thornhill Research Inc. Toronto, Canada) to deliver carefully controlled gas mixtures has been described in detail previously.[15] In brief, the basic function of the sequential rebreathing circuit (fit to the study subject) is to effectively limit the inspired gas to that of the output from the gas blender, independent of actual minute ventilation. This occurs because at any minute ventilation that exceeds the flow of gas from the blender the balance of inhaled gas is provided by previously exhaled gas from a circuit reservoir. The previously exhaled gas does not contribute to gas exchange as it has already equilibrated with the arterial blood. The flows and component gas concentrations of the gas entering the breathing circuit are calculated to target specific end-tidal CO_2_ and O_2_ tensions using algorithms described by Slessarev et al.[15] The timing and sequence of target end-tidal CO_2_ and O_2_ are prospectively entered into the MPET controller, which then implements them. With this system, the exhaled CO_2_ values have been shown to precisely reflect those in the arterial blood,[16] the true independent variable for brain blood flow. This system is capable of generating highly reproducible square wave and ramp changes in CO_2_ under constant O_2_ tensions as used here (henceforth when discussing the gas mixtures programmed by the MPET we will note these as CO_2_ or O_2_ but imply end-tidal partial pressure of CO_2_ or O_2_ unless otherwise stated).

### MRI sequences

MRI was performed on a 3.0-Tesla scanner with an 8-channel phased array head coil. T1-weighted anatomic images were acquired using a 3-dimensional spoiled gradient echo pulse sequence (whole brain coverage; matrix: 256×256; slice thickness: 2.2 mm; no interslice gap). BOLD MRI data were acquired with a T2*-weighted single-shot gradient echo pulse sequence with echo planar readout (field of view: 24**×**24 cm; matrix: 64**×**64; TR: 2000 ms; TE: 30 ms; flip angle: 85°; slice thickness: 5.0 mm; interslice gap: 2.0 mm; number of frames: 254).

### Post-hoc Analysis Patient Group: CVR maps

**Figure 1:**
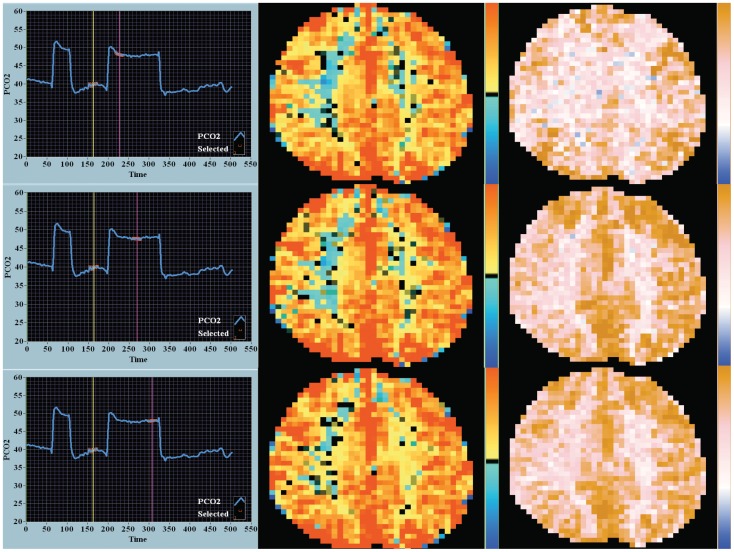
Response to square wave CO_2_ stimulus – CVR and DFA maps. These data are from a patient who underwent the square wave sequence and ramp sequence (Figure 2) in the same sitting. Not shown in this image is the end-tidal O_2_ tension which is stable over time at normal values (approximately 100 mmHg). The CO_2_ stimulus is shown in the first column – start, middle and end during the two-minute stimulus. The first 45-second square wave pulse was to aid in time sequencing of the BOLD signal and CO_2_ stimulus. Time is on the x-axis and end-tidal CO_2_ in mmHg on the y-axis. The yellow and magenta vertical markers highlight the center of the CO_2_ durations analyzed in the square wave sequence. The highlighted red lines are the points where the CO_2_ tension was correlated to the MR–BOLD signal for the CVR analysis. The second column shows the corresponding CVR maps. The color key to the right of the image is upper red +0.56 arbitrary BOLD units/mmHg in CO_2_ tension – deep blue −0.56. The third column shows the corresponding DFA maps. For the DFA maps the BOLD signal analysis was based on output as interpolated between the yellow and magenta markers. The color key to the right of the image is brown – 1.5 Hurst units, pink – 1.0 Hurst units, white – 0.5 Hurst units and deep blue – 0 Hurst units. ‘Blue’ DFA was defined as less than 0.5 Hurst units and indicates anti-persistent noise. The percentage of ‘blue’ DFA voxels noticeably decreased over time. The step change in end-tidal CO_2_ is 8 mmHg.

**Figure 2:**
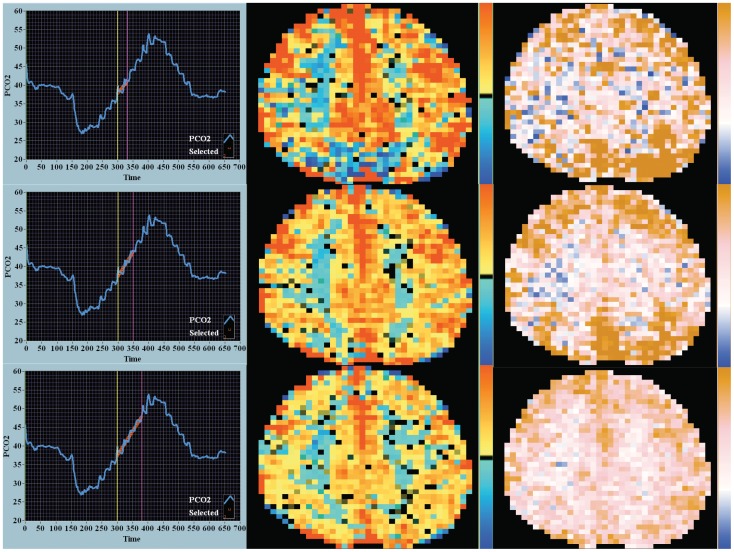
Response to ramp CO_2_ stimulus – CVR and DFA maps. The CO_2_ ramp stimulus is shown in the first column. The duration of the ramp stimulus increases by row. Not shown in this image is the end-tidal O_2_ tension which is stable over time at normal values (approximately 100 mmHg). The yellow and magenta vertical markers highlight the center of the CO_2_ durations analyzed in the square wave sequence. The highlighted red lines demonstrate the interpolated points where the CO_2_ tension was correlated to the MR–BOLD signal for the DFA analysis. The CVR maps are shown in second column and the DFA maps in the third column. In the first time period CVR map (row 1, column 2) the deep saturation of red signal indicates initial very high responsiveness (steep slope) within these areas suggesting these voxels are on the steep linear portion of the sigmoidal CO_2_ response curve. In the DFA map (row 1, column 3) these areas are noticeably brown with a Hurst exponent of 1.5 indicating very high time memory. The ramp change in end-tidal CO_2_ examined is 8 mmHg. The color keys are as in Figure 1.

Post-processing of the BOLD signal was per the usual techniques employing standard analysis of functional neuroimages (AFNI).[17] Custom-designed software time sequenced the CO_2_ alterations to the BOLD output. Here CVR is defined as the change in BOLD signal from a mean baseline measurement to a mean measurement at the altered CO_2_/mean change in CO_2_ tension between the two measurement periods. A full description is given in reference 13. The CVR was thresholded from −0.56 to +0.56 arbitrary BOLD units/mmHg change in CO_2_. Additionally, the raw MR output was imported into custom-written software (LabVIEW, National Instruments, Austin, TX). The CVR analysis was based on a two minute square wave alteration in CO_2_ tension from 40–50 mmHg. ‘Blue’ CVR maps – a decrease in BOLD signal with the CO_2_ stimulus (threshold 0 to −0.56) were quantified as the percent of total voxels for the axial slice. Three time periods were chosen at the start, middle and end of the sequence. The axial slice with the greatest number of voxels was chosen for analysis in each case. In the patient undergoing both square wave and ramp CO_2_ protocols sequentially, the ramp data were analyzed in an incrementally increasing fashion over the same CO_2_ range – see Figures 1 and 2 respectively.

### Post-hoc Analysis Patient Group: DFA maps

DFA is a temporal fractal analysis approach. The raw BOLD signal sequenced to the CO_2_ alterations noted above were analyzed using custom-written software (LabVIEW). The *interpolated* time periods of those used in the CVR maps were analyzed. DFA is a modified root mean square analysis of the magnitude of the noise signal fluctuations based on the granularity of the data (the time series is divided into boxes of equal length, with the longer box length customarily associated with greater signal fluctuation). A comprehensive description of the technique in relation to BOLD signal processing is given by Hu et al.[14] More generalized introductions to the technique are given by West [18] and Seely and Macklem.[19] A log-log plot of the relationship between the magnitude of fluctuation with increasing box length versus the box length is calculated. If the log-log transform of these data points have linear characteristics, the slope of this line is designated the Hurst exponent. The magnitude of the Hurst exponent is associated with well described noise patterns that have historically been identified with colors. Data were analyzed with a threshold for the Hurst exponent from 0 to 1.5. The color coding of the DFA maps were as follows – brown for a Hurst exponent in the range of 1.5 (Brownian noise), pink for a Hurst exponent of 1.0 (pink noise), white for a Hurst exponent of 0.5 (white noise) and blue for a Hurst exponent of 0 (anti-correlated noise). ‘Blue’ DFA maps – an anti-correlated time course of the BOLD signal with CO_2_ stimulus (threshold 0.5 to 0.0) were quantified as percent of total voxels for the axial slice. Such ‘blue’ maps were compared and contrasted to the ‘blue’ CVR maps described above.

### Post-hoc Analysis Volunteer Subject Group

The data were recorded and stored using a custom-designed digital acquisition system (LabVIEW). Data were analyzed following download to spreadsheet and time sequenced. The various hemodynamic responses to the same ramp sequence used in the MRI studies were analyzed. Heart rate, mean arterial pressure, middle cerebral artery velocity, and frontal cerebral oximetry responses were correlated to the change in CO_2_ generated by the MPET gas blender.

### Statistical Analysis

Data were analyzed using XLStat. Repeated measures ANOVA was used to compare CVR to DFA over the 3 time periods studied; p<0.05 considered significant by Tukey's test. Histogram analysis was fit to a maximal bin number of 25 for both CVR and DFA output. Correlations between hemodynamic variables versus CO_2_ changes were assessed by linear regression analysis; p<0.05 considered significant.

## Results

The demographics of the 8 patients with carotid stenosis are shown in Table 1. Angiographically documented severe cerebrovascular stenosis or occlusion was seen in all patients.

**Table 1:** Patient Demographics.

Patient	Age	Sex	Angiographic Findings
1	76	F	Bilateral ICA stenosis
2	60	F	Right ICA occlusion
3	48	F	Right ICA occlusion
4	35	F	Left ICA occlusion
5	53	M	Left ICA stenosis
6	69	F	Right ICA stenosis
7	70	M	Right ICA stenosis, Left ICA occlusion
8	70	F	Bilateral ICA stenosis

ICA – internal carotid artery.

A comparison of CVR maps with DFA maps over time in one patient where both the square wave and ramp sequences were performed sequentially in one sitting are shown in Figures 1 and 2. The leftmost panels in each figure show the alterations in CO_2_ (square wave – Figure 1 or ramp – Figure 2). The middle columns in each Figure show the CVR maps and the rightmost column the DFA maps (see the accompanying commentary with the Figures for a description of differences). Visual inspection indicates that the ‘blue’ voxel counts resolve more rapidly over time with DFA. Also of note is the greater percentage of ‘blue’ voxels with the ramp protocol, especially at the outset, but, as in the square wave CO_2_ challenge, resolution occurs in the later images. Also in the ramp sequence note the intense red saturation in the CVR map and the bleaching over time and the ‘brown’ saturation of high signal in the corresponding DFA map.

**Figure 3:**
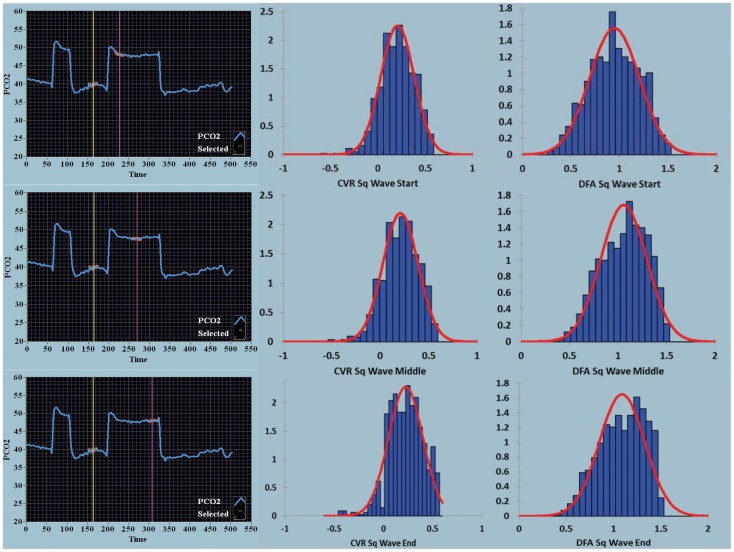
Density histograms over time for the square wave stimulus for CVR and DFA maps. The square wave sequence is shown in the first column, as defined in Figure 1. The second column shows the density histograms for the CVR output range shown on the x-axis (−0.56–0.56 BOLD units/mmHg CO_2_). The third column shows the density histograms for the DFA output range shown on the x-axis (0–1.5 Hurst exponent units). The red bell-shaped curves seen with the histograms are best normal curve fit to the histogram data. For the square wave sequences from start, middle and end, the mean and coefficient of variation for the CVR maps were 0.207, 0.855; 0.209, 0.871; and 0.227, 0.175 respectively, and for the DFA maps 0.947, 0.269; 1.06, 0.224; and 1.09, 0.222.

**Figure 4:**
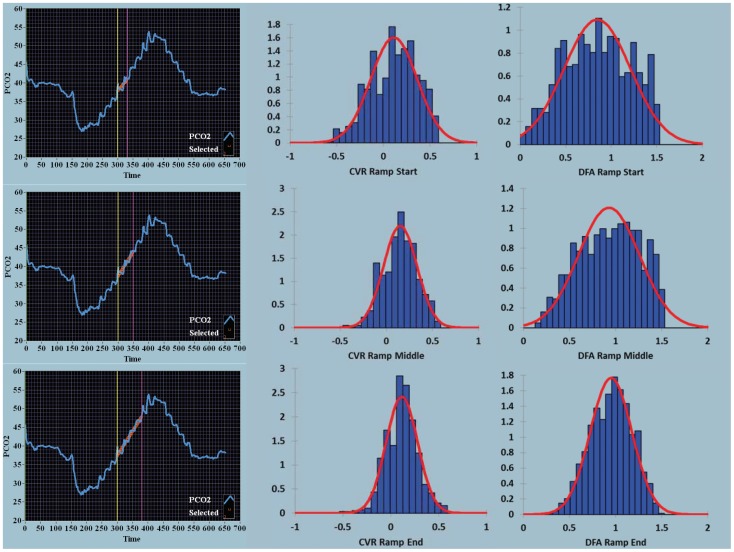
Density histograms over time for the ramp stimulus for CVR and DFA maps. For the ramp sequences from start, middle and end, the mean and coefficient of variation for the CVR maps were 0.113, 2.203; 0.151, 1.197; and 0.116, 1.422 respectively and for the DFA maps 0.847, 0.432; 0.929, 0.355; and 0.951, 0.237. Same color coding as in Figure 3.

The density histograms collating the voxel-by-voxel output for each image as seen in Figures 1 and 2 are quantified in Figures 3 and 4 respectively. With both the square wave (Figure 3) and ramp (Figure 4) sequences there was a decrease in the coefficient of variation over time seen for the CVR and DFA maps. With DFA the maximal density in the histogram, in this one patient, peaked near a Hurst exponent of 1 – indicating centering at pink noise.

**Figure 5:**
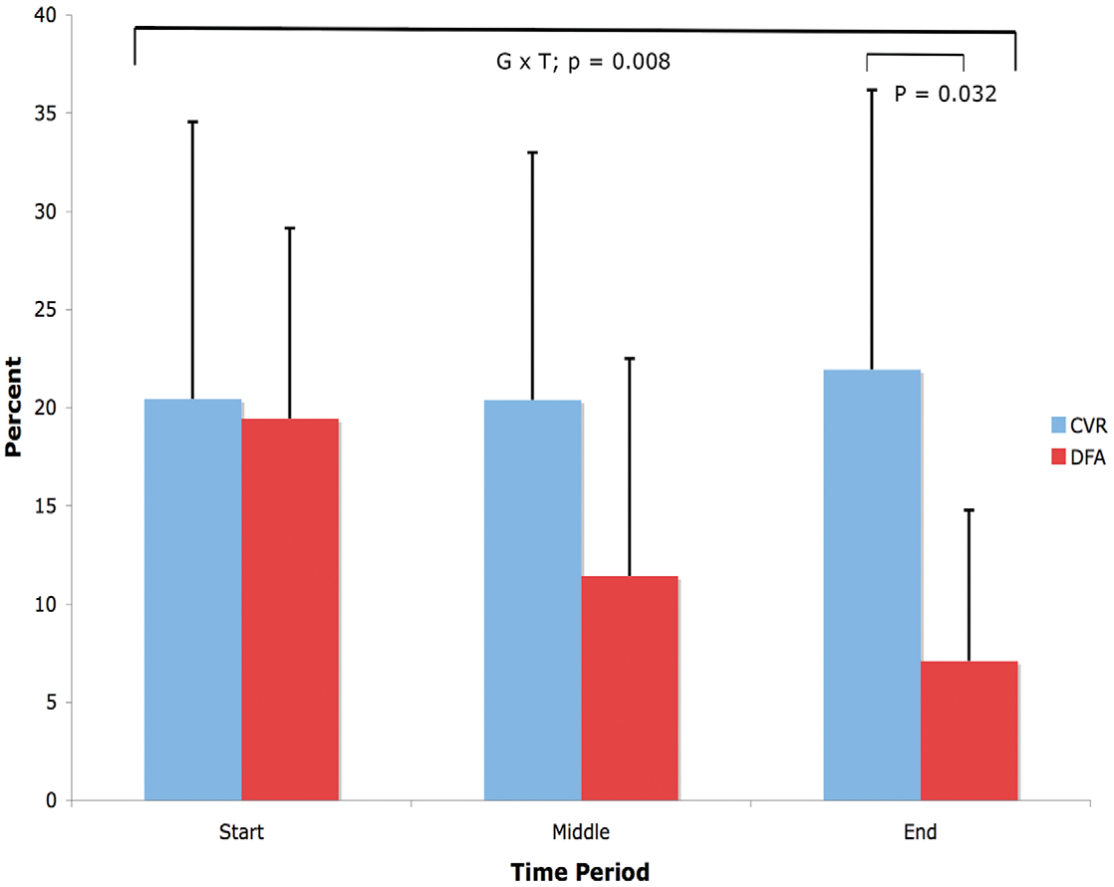
Change in percentage of ‘blue’ voxels over time in CVR and DFA maps. Mean percentage of ‘blue’ voxels for the CVR (0 to −0.56 BOLD units/mmHg CO_2_) and DFA (0.5 to 0 Hurst exponent units) maps for the 8 patients with carotid steno-occlusive disease. All patients had square wave CO_2_ stimuli. A significant difference in mean percentage of ‘blue’ voxels was seen between CVR and DFA for the end time period.

Figure 5 shows results from a repeated measures ANOVA for data from the 8 patients imaged with steno-occlusive disease of their carotid arteries. A significant group × time interaction for percentage of ‘blue’ voxels over time is seen with p = 0.008. Within group comparisons reveal that the percentage of ‘blue’ voxels is significantly less with DFA compared to CVR at the end measurement period (p = 0.032 by Tukey's test).

**Figure 6:**
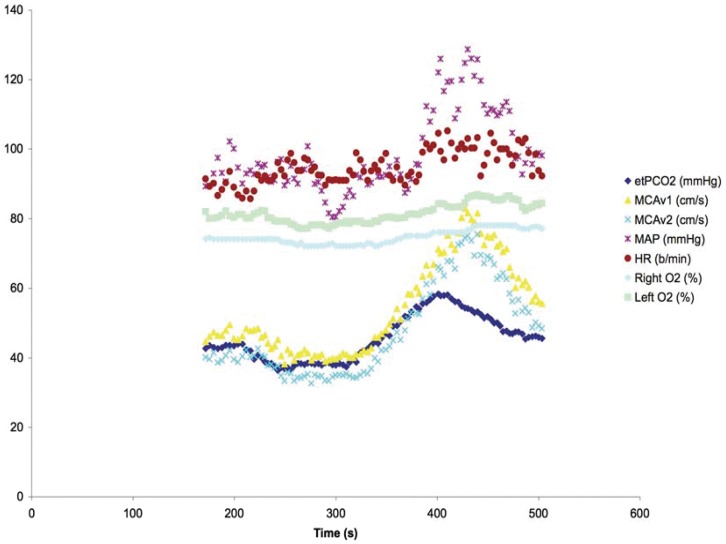
Vigorous hemodynamic response to CO_2_ ramp stimulus. Hemodynamic response to ramp CO_2_ stimulus in a 60 year old healthy subject. Note the marked blood pressure and cerebral blood flow velocity response to increased CO_2_ persisting past the stimulus peak. Legend abbreviations – etPCO_2_; end-tidal partial pressure of carbon dioxide, MCAv; middle cerebral artery velocity, MAP; mean arterial pressure, HR; heart rate, right and left O_2_; cerebral O_2_ saturation.

**Figure 7:**
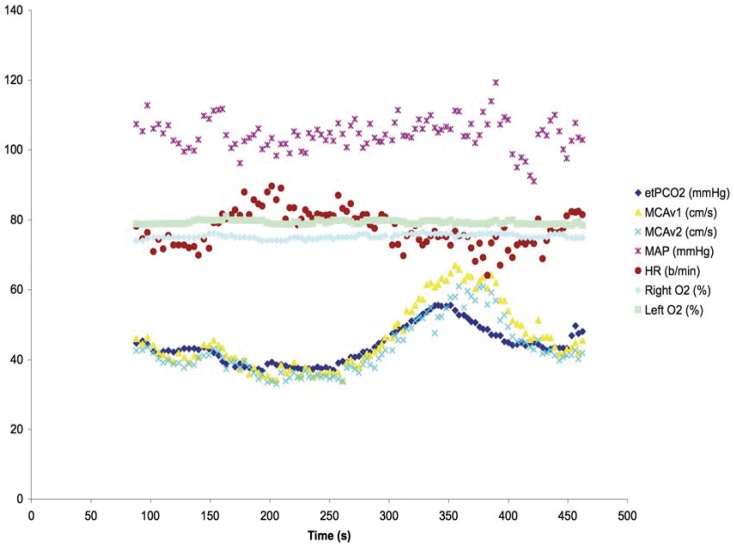
Muted hemodynamic response to CO_2_ ramp stimulus. Hemodynamic response to ramp CO_2_ stimulus in a 31 year old healthy subject. A much-attenuated response is seen in this individual. Same legend abbreviations as in Figure 6.

Figure 6 shows the hemodynamic effects on a 60 year old subject undergoing the same ramp sequence as used for the patient highlighted in Figure 2. A marked hyperdynamic blood pressure and cerebral blood flow velocity response is seen with increasing CO_2_ that persists beyond the application of the CO_2_ stimulus. By contrast, Figure 7 shows the hemodynamic effects in a 31year old subject with the ramp sequence; comparatively a much-dampened response is seen. Furthermore there is no increase in cerebral oxygen saturation to CO_2_ stimulus.

### Supporting Information – Movie S1

The accompanying movie shows the dynamic change in CVR calculated from the alteration in BOLD signal response to ramp changes in CO_2_ tension over time.

## Discussion

This study examines dynamic BOLD imaging of the brain and response to computer-controlled, repeatable CO_2_ stimuli. The potential to quantify the cerebrovascular responsiveness and time course in patients at serious neurological risk from ischemic events can be appreciated. The use of two approaches to initiate a controlled change in CO_2_ (square wave and ramp stimuli) and two analysis techniques of the BOLD signal (CVR and DFA) highlight how the brain regionally responds to changes in CO_2_ that cannot be gleaned solely by one approach or one analysis technique. Our analysis of the dynamic response to the ramp stimulus indicates both similarities and differences to that seen with the more commonly reported square wave stimulus. We note that the square wave challenge provides a window into the dynamic effect of time on the cerebral vasculature response to a constant stimulus. Whereas, by contrast, the ramp challenge highlights the effect of increasing CO_2_ alone as time is a controlled variable as the rate of change of the increasing CO_2_ signal is constant. The dynamics of the response to CO_2_ can be appreciated by examination of the first measurement period in the ramp sequence (row 1 in Figure 2). The intense red signal in the CVR map (middle column) and brown signal in the DFA map (right column) suggest that these voxels are demonstrating a CO_2_ response centered about the steep linear portion of the sigmoidal cerebrovascular response curve at a CO_2_ of 40 mmHg. On this portion of the CO_2_ response curve cerebral blood flow velocity can change as much as 11percent/mmHg increase in CO_2_ tension.[2] The color fading seen in the latter two periods (rows 2 and 3) indicate a diminished responsiveness (a shallower slope) due to incorporation of BOLD signal averaging while on the plateau of the flow response to increases in CO_2_ tension.

The generation of CVR maps using square wave CO_2_ stimuli with these techniques have been previously reported.[20] [21] Important therapeutic decisions have been based on longitudinal imaging in patients [7]; the reproducibility of the CO_2_ stimulus allows such comparisons to be reliably made.

Detrended fluctuation analysis (DFA) is one approach to fractal signal processing.[18] [22] The technique has been applied successfully to functional MRI signal analysis.[14] The application of DFA to BOLD signal analysis is predicated on the premise that the technique is able to identify whether or not the signal examined has a ‘time memory’. White noise has no memory – the signal is truly random – and has a Hurst exponent of 0.5 when the signal is processed by DFA. In contrast, many healthy physiological signals have a Hurst exponent greater than 0.5 and often close to 1.0 – pink noise. This is noise with a memory. The greater exponent indicates a repetitive pattern to the signal at every scale.[23] In the functional MRI study referenced activated voxels often demonstrated a Hurst exponent near unity. The interpretation is that the repetitive stimulus – say finger tapping – increases the BOLD signal in a reproducible manner during the course of the stimulus. The BOLD signal which is inherently noisy now has its noise altered by the superimposed stimulus – noise now with a memory of the activation initiated by the finger tapping. Examination of our data indicates that a reproducible CO_2_ stimulus induces a robust noise memory – so much so that the Hurst exponent approached or exceeded 1.5 – Brownian noise. In a novel manner we also examined the distribution of ‘blue’ voxels with a Hurst exponent of less than 0.5. This exponent is defined as being associated with anti-correlated or anti-persistent noise and we interpret these findings as indicating a decreasing BOLD signal with increasing CO_2_ – akin to the negative CVR slope indicating cerebral flow redistribution or steal depicted by ‘blue’ CVR voxels.

The DFA findings suggest that this technique also provides robust maps correlated to changes in BOLD signal intensity. The intensity of this signal is likely most influenced at the venular level.[24] The oxygen saturation of venous hemoglobin, venous volume, and metabolic rate can all influence the signal intensity, and recent work indicates that altered diffusion can also influence BOLD signal strength.[25] DFA demonstrates a dynamic response as opposed to CVR, which is a more static ratio of net signal change to the change in CO_2_. DFA is calculated from raw signal over time, while the signal for CVR is a meaned value over time at different CO_2_ levels. As such, the changes in BOLD signal as measured by DFA may be tracking dynamic changes in oxygen extraction fraction from the blood. Consistent with this hypothesis is the finding of an increase in signal memory (increasing Hurst exponent) with activated voxels in functional MRI studies as a surrogate for increased venous oxygenation (and reduced oxygen extraction fraction). In an analogous fashion, the increase in anti-correlated DFA signal (decreasing Hurst exponent) seen in the ‘blue’ voxels should map decreased venous oxygenation or an increased oxygen extraction fraction. Follow-up studies relating ‘blue’ DFA maps to quantitative BOLD imaging or PET imaging with ^15^O may permit more definitive interpretation.[26]

Attenuation of the ‘blue’ DFA signal over time in both the square wave and ramp CO_2_ sequences requires comment. A partial explanation may be surmised from the two studies done in the subjects with measures of cerebral oxygen saturation using near infrared spectroscopy and middle cerebral blood flow velocity with transcranial Doppler (Figures 6 and 7). In both these studies, cerebral blood flow velocity (as a surrogate for cerebral blood flow) showed a progressive increase in flow velocity that outlasted the maximal CO_2_ response of the ramp sequence. In the first subject (Figure 6) there was also a vigorous increase in blood pressure to the CO_2_ stimulus. In the presence of increased CO_2_ the cerebral circulation becomes pressure passive and cerebral blood flow would passively increase with increased driving pressure. The dynamic responsiveness, with resolution of ‘blue’ DFA voxels over time may be, in part, contingent on the systemic pressure mediated changes in response to CO_2_. Thus, to the extent that risk is related to either the regional changes in blood flow or its time course, the interplay between CVR and DFA responsiveness provides two windows from which to view individual patient neurological risk in the presence of steno-occlusive disease of the cerebral vasculature.

With DFA the histogram density peaked at a Hurst exponent near 1.0 with a decrease in the coefficient of variation. Healthy physiologic signals often are seen with a Hurst exponent approaching unity. Highlighting the one patient with both a square wave and ramp sequence suggests an adaptive response is seen to the perturbation of CO_2_ stimulus. One interpretation from this patient is that these results support the concept of allometric control in response to a stimulus – measuring the fractal time signature of the signal response and its change over time.[23] West and colleagues have also advanced the notion of maximal information transfer in fractal physiological systems when signal noise is tuned to a fractal exponent of unity.[27] [28]

In summary, we present two protocols to induce a CO_2_ stimulus and two analysis techniques to process the resultant MR-BOLD signal. Such an approach demonstrates the potential utility of an enhanced brain stress test in the evaluation of patients with cerebrovascular steno-occlusive disease. Further studies based on these findings may delineate which patients are at greatest risk of stroke or post-operative delirium – to name but two examples – following surgical procedures.

## Supporting Information

Movie S1 **Changes in CVR over time with the ramp stimulus in a patient with ‘blue’ brain.** This video shows the alterations in CVR slope with ramp alterations in end-tidal CO_2_ tensions. The dynamic behavior is in evidence here over the course of the provocative stimulus.
(MP4)
